# Pellino 1 inactivates mitotic spindle checkpoint by targeting BubR1 for ubiquitinational degradation

**DOI:** 10.18632/oncotarget.16762

**Published:** 2017-03-31

**Authors:** Jihyun Park, Hye-Young Park, Suhyeon Kim, Hyun-Soo Kim, Ji Y. Park, Heounjeong Go, Chang-Woo Lee

**Affiliations:** ^1^ Department of Health Sciences and Technology, SAIHST, Sungkyunkwan University, Seoul 06351, Republic of Korea; ^2^ MOGAM Institute for Biomedical Research, Yongin 16924, Republic of Korea; ^3^ Department of Molecular Cell Biology, Sungkyunkwan University School of Medicine, Suwon 16419, Republic of Korea; ^4^ Department of Pathology, Daegu Catholic University Medical Center, School of Medicine, Catholic University of Daegu, Daegu 42472, Republic of Korea; ^5^ Department of Pathology, University of Ulsan College of Medicine, Asan Medical Center, Seoul 05505, Korea

**Keywords:** Pellino 1, BubR1, receptor-mediated signalling, mitotic spindle checkpoint, aneuploidy

## Abstract

Aberrant constitutive activation of receptor-mediated downstream signalling plays an active role in the deregulation of cell cycle control. The mitotic spindle checkpoint is important in preventing abnormal mitotic cell cycle with chromosome missegregation from achieving neoplastic aneuploidy. However, mechanisms coupling receptor-mediated signalling to mitotic spindle checkpoint regulation remain unclear. Pellino 1 is a receptor signal-responsive E3 ubiquitin ligase, and the application of certain receptor-mediated signalling regulates the expression and activity of Pellino 1. In the present study, Pellino 1 expression induced extensive chromosome aneuploidy and allowed abnormal mitotic cells to adapt and become aneuploid *in vitro* and *in vivo*. Pellino 1 directly interacted with BubR1, a key component of mitotic spindle checkpoint, in a mitotic cell-cycle dependent manner, and down-regulated the stability of BubR1 by ubiquitination-mediated degradation and induced mitotic dysfunction. In summary, Pellino 1 expression acts as an inhibitory signal of the homeostatic regulation of mitotic cell cycle and checkpoint, and thus contributes to the initiation and progression of neoplastic chromosome aneuploidy.

## INTRODUCTION

Pellino 1 (Peli1) is a signal-responsive E3 ubiquitin ligase that promotes the K48-linked polyubiquitination of several targets, including c-Rel for ATP-dependent proteolysis by the proteasome [1], and the K63-linked ubiquitin chains that mediate nonclassical, degradation-independent modification, such as activation of TRAF-6 [2, 3]. Interestingly, the deficiency of Peli1 leads to hyperactivation and nuclear accumulation of c-Rel in response to T-cell receptor (TCR)-CD28 signalling, which contributes to the development of autoimmune diseases like experimental autoimmune encephalomyelitis, and is also associated with impaired toll-like receptor (TLR) 3-and TLR4-induced activation of nuclear factor-kappa B (NF-kB) and reduced expression of proinflammatory genes [1, 4]. In addition, evidence from Pellino 3-deficient mice has revealed that Pellino 3 is dispensable for TLR-induced expression of proinflammatory cytokines, and has a negative regulatory role in TLR3- and virus-induced expression of type 1 interferons and related genes. Interestingly, the application of TLR signalling, such as treatment with TLR3 or TLR4 agonists, activates the expression and E3 ligase activity of Peli1 in B and T cells [1, 4, 5, 7], indicating the important role of Pellino proteins in regulating the proliferation and activation of immune cells in responding to receptor-mediated signalling.

Mice that overexpress Peli1 have several distinct phenotypes depending on the expression level of Peli1. Overexpression with a high level of Peli1 is associated with severe rates of mortality and chromosome instability (our unpublished observations), whereas the constitutive overexpression of a relatively low level of Peli1 leads to the development of a wide range of lymphoid tumours and a shortened lifespan for the animals [6]. However, how the unbalanced accumulation or activity of Peli1 can contribute to the increased risk of the manifested diseases remains unknown. Interestingly, Peli1 expression is highly attenuated in normal immune and myeloid cells, but is activated in response to signalling mediated by various receptors [1, 4, 5]. Collectively, it is becoming increasingly clear that Peli1 expression and activity are highly suppressed in normal or non-pathological conditions, and/or are strictly limited in a tissue-specific manner and are subjected to differential regulation by a specific signalling pathway.

Recent studies have indicated that receptor-mediated signalling is closely linked with cell cycle progression and checkpoint controls [8]. As one of these control points, the mitotic spindle checkpoint ensures accurate chromosome segregation by delaying anaphase onset until all chromosomes have properly attached to the mitotic spindle. Activation of the mitotic spindle checkpoint is facilitated by the binding of BubR1, Bub3 and Mad2 to Cdc20 to prevent activation of the anaphase-promoting complex/cyclosome (APC/C; an inhibitory E3 ubiquitin ligase) until all chromosomes have achieved bipolar kinetochore-microtubule attachment [9, 10]. In particular, BubR1 inhibits the ubiquitination activity of APC/C by blocking formation of the active Cdc20-associated APC/C (APC/C^Cdc20^) complex [9, 11]. BubR1 also interacts with the kinetochore motor protein, CENP-E [12–14]. Importantly, BubR1 appears to be involved in the proper attachment of microtubules to the kinetochores, thereby linking the regulation of chromosome-spindle attachment to mitotic checkpoint signalling [15–18]. However, there is no clear evidence on how mitotic spindle checkpoint signalling is deregulated in disease development.

Mutational inactivation or disruption of BubR1 activity can result in loss of checkpoint control, chromosomal instability (caused by premature anaphase) and/or early onset of malignancy [19–22]. Germline mutations in the BUB1B gene encoding BubR1 cause premature chromatid separation and lead to the mosaic variegated aneuploidy syndrome, which is characterized by constitutional aneuploidy and a high risk of childhood cancers [23]. In addition, BubR1 functions as a potent apoptotic molecule and prevents abnormal mitotic cells with chromosomal instability from adapting [24]. Together, these reports suggest that by fine-tuned regulation of the mitotic spindle checkpoint, BubR1 is an important inhibitory factor in acquisition of preneoplastic aneuploidy and development of cancer.

In this study, we provide the first molecular evidence that Peli1, a key receptor signalling adaptor molecule, directly deregulates the mitotic spindle checkpoint by interacting with BubR1 for ubiquitination-mediated degradation. Therefore, it is likely that the forced activation of receptor downstream signalling cascade by Peli1 expression leads to severe impairment of the mitotic spindle checkpoint, thus contributing to production of chromosome aneuploidy and cancer.

## RESULTS

### Peli1 induces extensive chromosome aneuploidy *in vivo*

**Figure 1:**
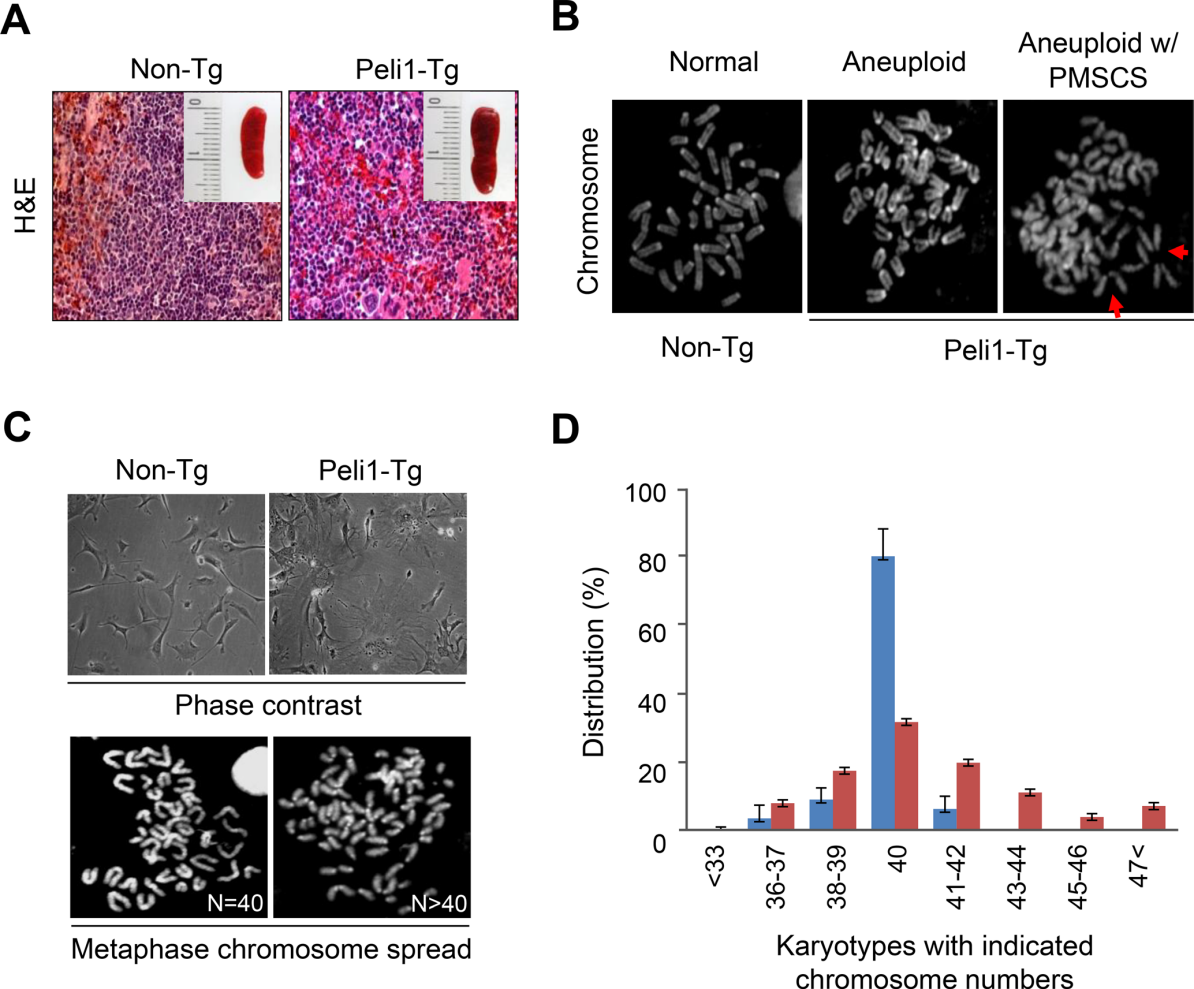
Transgenic mice overexpressing Peli1 induce aneuploidy and develop tumours (**A**) Representative microscopic images of hematoxylin and eosin-stained spleens showing normal and lymphoma phenotypes in non-Tg littermate and Peli1-Tg mice, respectively. (**B**) Mitotic chromosomes of non-Tg and Peli1-Tg splenocytes were spread and visualized by Giemsa staining. Values shown are the percentages of aneuploid populations (> 110 splenocytes were counted per experiment). PMSCS, premature sister chromatid separation (**C**) Representative phase-contrast images (upper panel) and mitotic chromosomes (lower panel) of non-Tg and Peli1-Tg mouse embryo fibroblasts (MEFs). (**D**) Chromosomes of non-Tg and Peli1-Tg MEFs were spread and visualized by Giemsa staining. Values represent the mean percentages of chromosome numbers determined from three independent experiments (> 200 cells were counted per experiment).

Since the overexpression of Peli1 contributes to development of B cell lymphoma and autonomous tumours in several organs [6], we questioned whether or not Peli1 expression could affect cellular functions, such as the cell cycle, cell death, differentiation, senescence and/or maintaining chromosome integrity. As Peli1-transgenic (Tg) mice develop tumours in various organs including the spleen, splenocytes were isolated from Peli1-Tg mice that displayed splenic plasmacytoid differentiation and lymphomas, and from control littermate non-Tg mice that had normal splenic features (Figure 1A, 1B). The metaphase chromosome-spreading assay revealed that chromosome aneuploid populations were significantly higher in Peli1-expressing splenocytes (50 ± 8.3%) compared with control splenocytes (19.1 ± 3.5%) (Figure 1B). In addition, premature sister chromatid separation (PMSCS), a hallmark of a defective mitotic checkpoint [19, 25], was observed in approximately 18 ± 1.7% of splenocytes isolated from Peli1-Tg mice but in < 5% of control splenocytes (Figure 1B). Furthermore, we isolated murine embryonic fibroblasts (MEFs) from non-Tg and Peli1-Tg mice (Figure 1C). Similarly, MEFs overexpressing Peli1 led to extensive chromosome aneuploidy, even prior to immortalization, compared to the control MEFs (Figure 1C, 1D). The Peli1-expressing MEFs displayed much larger increases in cell size (Figure 1C), implying that Peli1 expression acts as a causative factor for acquisition of aneuploidy *in vivo*.

### Peli1 directly interacts with BubR1 and their interaction is dependent on the mitotic cell cycle

**Figure 2:**
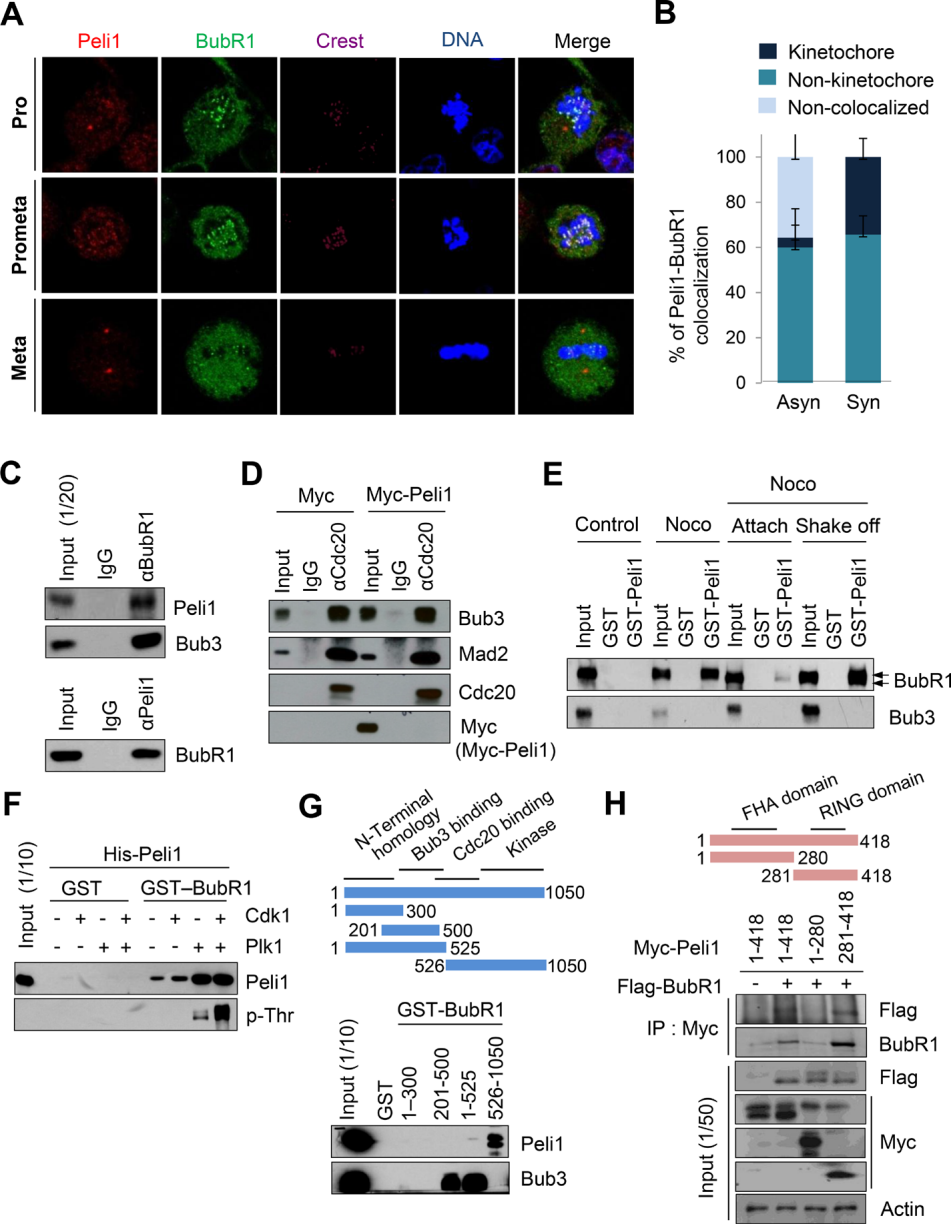
Peli1 co-localizes and interacts with BubR1 at mitotic phase of cell cycle (**A**) Immunofluorescence images of HeLa cells at the mitotic phase are shown. Cells were stained with anti-BubR1 antibody (green), anti-Peli1 antibody (red) or Hoechst (blue), and examined by confocal microscopy. (**B**) The graph represents the percentages of Peli1 co-localized with BubR1 at kinetochores in asynchronized or synchronized cells. At least 30 of each cell type were analysed in three independent experiments. Data are presented as mean values (*n* = 3). (**C**) Ramos cells were synchronized by treating nocodazole for 18 hr. Cell lysates were then immunoprecipitated with anti-BubR1 (upper) or anti-Peli1 antibody (lower) and then immunoblotted with anti-Peli1 and anti-Bub3 (positive control) or anti-BubR1 antibody. (**D**) HeLa cells were transfected with Myc (control) or Myc-Peli1 expression plasmids. At 24 hr post-transfection, cells were treated with nocodazole (200 ng/ml) for 24 hr and harvested for immunoprecipitation with an anti-Cdc20 antibody. (**E**) HeLa cells were synchronised using nocodazole and then separated into attached (Attach) and floating populations by mitotic shake-off (Shake-off). Cell lysates from asynchronous (control) and synchronous HeLa cells were incubated with beads bound to GST or GST-Peli1. Upper arrowheads indicate the hyperphosphorylated form of BubR1. (**F**) Bead-bound GST and GST-BubR1 were reacted with the Plk1 or Cdk1/Cyclin B kinase in the presence of unlabelled ATP. GST-BubR1 proteins phosphorylated or left unphosphorylated *in vitro* were incubated with purified His-Peli1. *In vitro* phosphorylated GST-BubR1 was immunoblotted with anti-Peli1 and anti-phosphothreonine (p-Thr) antibodies. (**G**) Structural schematic of BubR1 showing its NH_2_-terminal homology and Bub3-binding, Cdc20-binding, and kinase domains. Ramos cells were synchronised using nocodazole, and lysates were incubated with GST alone or with a series of BubR1 deletion mutants fused to GST. Bound proteins were resolved by SDS-PAGE and immunoblotted with an anti-Peli1 or anti-Bub3 (positive control) antibody. (**H**) Structural schematic diagram of Peli1 showing N-terminal forkhead-associated (FHA) domain and C-terminal RING domain with the characteristic feature of the RING class of E3 ubiquitin ligases. HeLa cells were transfected with Myc-Peli1 (full-length) (amino acids 1-418) or deletion mutants (amino acids 1-280 or 281-418) in combination with Flag-BubR1 expression plasmids. At 24 hr post-transfection, cells were treated with nocodazole (200 ng/ml) for an additional 24 hr and harvested for immunoprecipitation with an anti-Myc antibody and immunoblotting with anti-Flag antibody.

To initially examine the molecular mechanism leading to chromosome aneuploidy by Peli1 overexpression, we monitored the expression profiles of a series of representative marker proteins involved in the signalling pathway for the mitotic spindle checkpoint, including Bub1, Bub3, BubR1, Mad1, Mad2, Cdc20, CENP-E, Mps1 and Rassf1a, using splenocytes isolated from Peli1-Tg mice and non-Tg littermates. Interestingly, a major difference between the control splenocytes and the Peli1-overexpressing splenocytes was a sharp reduction in levels of BubR1, which is one of key regulators of the mitotic spindle checkpoint (data not shown). Next, we monitored the subcellular distributions of Peli1 and BubR1 to identify the function of Peli1 during mitosis (Figure 2A, 2B). Immunofluorescence analyses revealed localization of Peli1 at spindle poles and kinetochores (Figure 2A) and also at the non-kinetochore regions (Figure 2A, 2B). In particular, about one-third of the Peli1 protein localized at the kinetochores and efficiently colocalized with BubR1 during prometaphase (Figure 2B). Although a relatively small population of Peli1 associated with BubR1 at the kinetochore, it seemed that Peli1 functionally cross-talks with BubR1 and negatively regulates the mitotic spindle checkpoint.

To verify BubR1-Peli1 interaction, extracts from B lymphoblastic Ramos cells with endogenous Peli1 protein expression were immunoprecipitated with an anti-BubR1 antibody and then immunoblotted with an anti-Peli1 antibody and vice versa. The co-immunoprecipitation experiments revealed the formation of a complex between BubR1 and both Peli1 and Bub3 (Figure 2C). To test the possibility that Peli1-BubR1 interaction may affect the formation of the MSC, HeLa cells with very low levels of endogenous Peli1 expression were transfected with Myc-tagged Peli1 expression plasmid. Binding to Cdc20 was analysed by immunoprecipitation from the HeLa lysates with anti-Cdc20 antibody and Western blot probing with anti-Myc antibody (Figure 2D). Peli1 expression did not affect the interaction of the other mitotic checkpoint proteins, Bub3 and Mad2, with Cdc20 (Figure 2D).

To determine whether the BubR1-Peli1 interaction was regulated depending on the mitotic cell cycle, asynchronized HeLa cells were treated with nocodazole followed by a mitotic shake-off to separate the synchronized cells into attached and floating populations (Figure 2E). The interaction between glutathione S-transferase (GST)-Peli1 and endogenous BubR1 was barely detectable in attached cells (considered to represent non-mitotic cells). However, the GST-Peli1 and BubR1 interaction was strongly apparent in the floating population of synchronized cells, most of which were arrested in (pro) metaphase. This indicated that the interaction between BubR1 and Peli1 was dependent on the mitotic cell cycle.

To examine whether activation of BubR1 affects the interaction with Peli1, an *in vitro* binding assay was performed. During mitosis, Plk1 and its priming kinase Cdk1 phosphorylates and activates BubR1 [26]. A GST-BubR1 fusion protein was incubated in a reaction with recombinant Cdk1/Cyclin B kinase and/or Plk1 in the presence of unlabelled ATP. The resulting *in vitro* phosphorylated GST-BubR1 and GST alone (control) proteins were then incubated with purified His-tagged Peli1. Binding of the His-tagged Peli1 to phosphorylated GST-BubR1 was analyzed by immunoblotting with anti-Peli1. Anti-phosphothreonine probing was used to confirm the phosphorylation of GST-BubR1 and a control for the labelled GST fusions (Figure 2F). Of note, GST-BubR1 phosphorylated by Plk1 showed a much stronger interaction for His-Peli1 than the non-phosphorylated GST-BubR1. However, there was no further augmentation of Peli1 interaction with the GST protein phosphorylated by both Cdk1 and Plk1.

For identification of the domain responsible for the BubR1-Peli1 interaction, a series of GST-BubR1 deletion mutants were made, purified and incubated with extracts from synchronised Ramos cells. As shown in Figure 2G, GST fusion of BubR1 COOH-terminal (amino acids 526–1050) fragment containing the kinase domain and some portion of the Cdc20-binding domain formed a complex with Peli1, whereas GST fusion of fragments containing central (amino acids 201–500) and NH_2_-terminal homology (amino acids 1–300) regions did not. As a positive control, the interaction between the BubR1 central region (amino acids 201–500) and its well-defined binding partner Bub3 was verified. Next, HeLa cells were transfected with plasmids expressing a control Myc-epitope, Myc-tagged full-length Peli1 (amino acids 1–418) or Myc-tagged Peli1 deletion mutant individually or in combination with the Flag-BubR1 expressing plasmid (Figure 2H). Co-immunoprecipitation assay revealed that Peli1 COOH-terminal fragment (amino acids 281–418) containing a RING domain formed the complex with BubR1, whereas the NH_2_-terminal fragment (amino acids 1–280) containing two FHA homologous domains did not. Together, these results indicate that C-terminal of Peli1 interacted directly with activated BubR1 in the mitotic cell cycle.

### Peli1 expression down-regulates the stability of BubR1 by ubiquitination-mediated degradation and induces mitotic dysfunction

**Figure 3:**
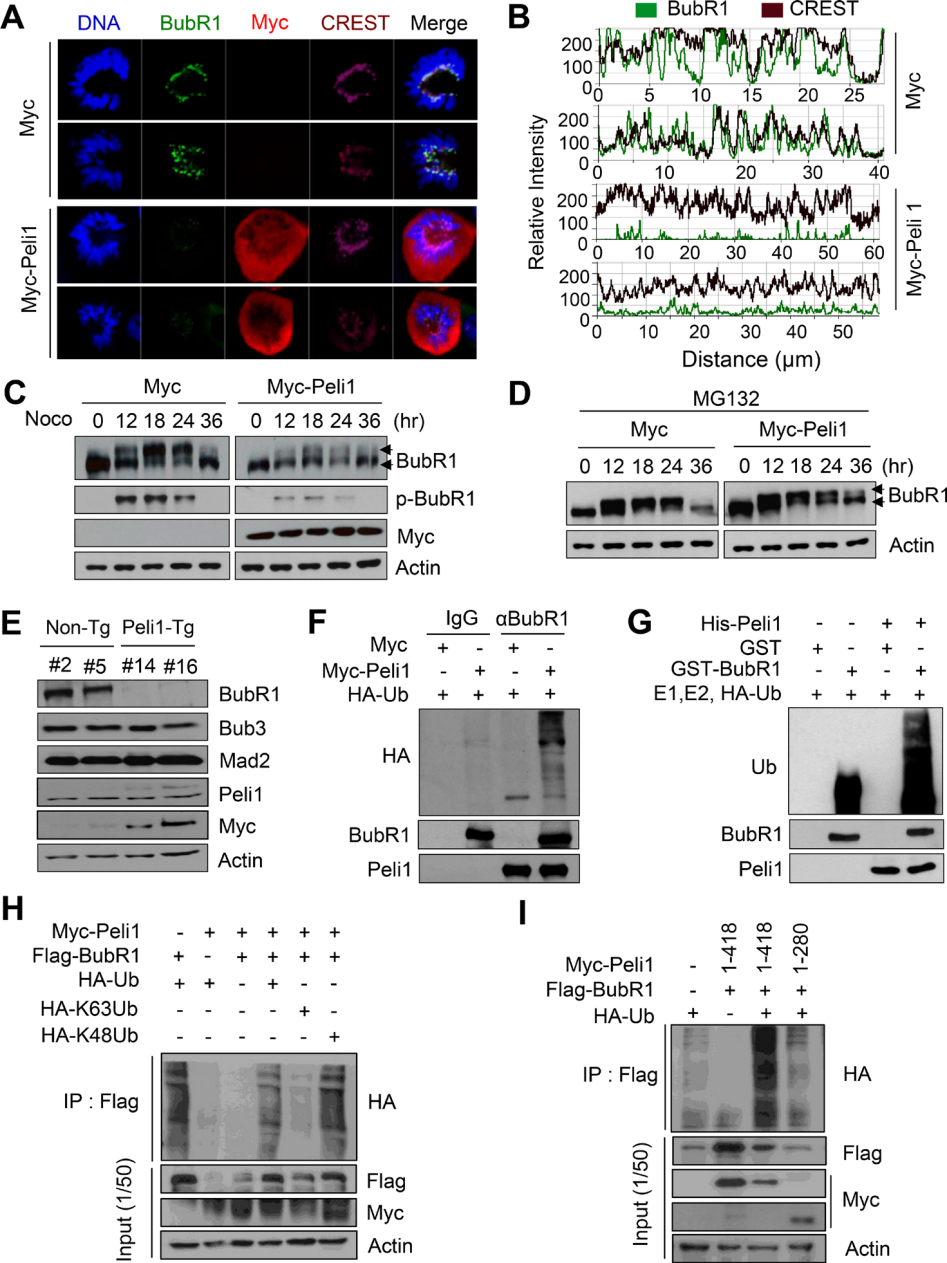
Peli1 directly down-regulates the stability of BubR1 by K48-mediated polyubiquitination (**A**) HeLa cells were transiently transfected with a Myc or Myc-Peli1 expression plasmid, stained with an anti-BubR1 (green), an anti-Myc (red) antibody, the CREST serum (purple), or Hoechst (blue), and examined by confocal microscopy. Results are representative of three independent experiments. (**B**) The relative intensity of BubR1 and proteins targeted by CREST serum was compared in control and Peli1-overexpressing cells. (**C**, **D**) HeLa cells were transfected with a Myc or Myc-Peli1 expression plasmid, at 24 hr post-transfection, treated with nocodazole for different time-points, and further cultured in absence or presence of 25 μM MG132 for 5 hr. Harvested cellular lysates were immunoblotted with an anti-BubR1 antibody. Upper arrowheads indicate the hyperphosphorylated form of BubR1. (**E**) Splenocytes were isolated from non-Tg and Peli1-Tg mice and stimulated by lipopolysaccharides. At 24 hr post-treatment, splenocytes were harvested, lysed, and subjected to immunoblotting with the indicated antibodies. (**F**) HeLa cells were cotransfected with Myc or Myc-Peli1 and an HA-ubiquitin expression plasmid and then cultured in presence of nocodazole and MG132 as described above. The BubR1 immunocomplex was immunoprecipitated from transfected cell lysates with an anti-BubR1 antibody and subjected to immunoblotting with anti-HA, anti-BubR1 and anti-Peli1 antibodies. (**G**) Purified GST or GST-BubR1 was incubated with purified His-Peli1 in conjunction with E1, E2, and hemagglutinin-tagged ubiquitin (HA-Ub) enzymes. Reaction mixtures were immunoblotted with anti-ubiquitin and anti-Peli1 antibodies. (**H**) HeLa cells were transfected with Myc-Peli1, Flag-BubR1 and HA-Ub, HA-K63Ub or HA-K48Ub expression plasmids and then cultured in presence of nocodazole and MG132. Cells were harvested for immunoprecipitation with anti-flag antibody and immunoblotting with anti-HA antibody. (**I**) HeLa cells were transfected with Myc-Peli1 (full-length) (amino acids 1-418) or C-terminal RING domain deletion mutant (amino acids 1-280) in combination with Flag-BubR1 and HA-Ub expression plasmids. Cells were then cultured in presence of nocodazole and MG132 and harvested for immunoprecipitation with anti-Flag antibody and immunoblotting with anti-HA antibody.

To test whether Peli1 as an E3 ubiquitin ligase affects the stability of BubR1, we examined the levels of endogenous BubR1 protein in cells overexpressing Peli1. HeLa cells with nearly undetectable levels of endogenous Peli1 protein were transfected with a Myc-Peli1 expression plasmid and then immunostained with anti-BubR1, anti-Myc antibodies and CREST serum (a marker for kinetochore proteins) (Figure 3A, 3B). Control cells clearly showed BubR1 immunostaining at the kinetochores, but Peli1 overexpression sharply reduced the kinetochore-localized BubR1 staining. However, Peli1 expression did not influence the levels of other mitotic checkpoint proteins, Bub1 and Mad2, at the kinetochores (Supplementary Figure 1B, 1C), or the interactions between Bub3 or Mad2 and Cdc20 (Figure 2D). To validate this data, cellular fractionation was performed with control or HeLa cells overexpressing Peli1 (Supplementary Figure 2A). Chromatin-bound BubR1 was downregulated in cells overexpressing Myc-Peli1 after synchronization by nocodazole. These results indicate that Peli1 expression specifically controlled BubR1 stability through a direct interaction during mitosis.

Next, we examined the levels of endogenous BubR1 protein in cells overexpressing Peli1. HeLa cells were transfected with a control or a Myc-Peli1-encoding expression vector and synchronised by nocodazole treatment. Levels of the endogenous BubR1 protein were compared. BubR1 is phosphorylated during mitosis, and this BubR1 phosphorylation is critical for both stable kinetochore attachment and checkpoint activation [24, 26]. As shown in Figure 3C, hyperphosphorylation of BubR1 was sharply increased after nocodazole treatment, and these elevated levels were maintained up to 24 hr after the nocodazole treatment. Peli1 overexpression sharply reduced the stability of the BubR1 protein, particularly the hyperphosphorylated BubR1, but not the hypophosphorylated form. In addition, depletion of Peli1 significantly increased the level of hyperphosphorlyated BubR1 (Supplementary Figure 2B, 2D). The level of BubR1 in Peli1-overexpressing cells showed a significant recovery through treatment with MG132, a proteosome inhibitor (Figure 3D), indicating that Peli1 expression regulates BubR1 stability through protein degradation.

The levels of the BubR1 protein in control and Peli1-expressing splenocytes isolated from non-Tg and Peli1-Tg mice were compared (Figure 3E). Importantly, the level of BubR1 was significantly lower in Peli1-overexpressing splenocytes compared with control splenocytes. However, the levels of other mitotic checkpoint proteins, Bub3 and Mad2, were constant and comparable in splenocytes isolated from both non-Tg and Peli1-Tg mice (Figure 3E), indicating that Peli1 specifically deregulated BubR1 but not other mitotic checkpoint proteins.

To assess whether the reduced stability of BubR1 by Peli1 expression was due to ubiquitin-dependent degradation, we reconstituted the ubiquitination assay for BubR1 protein. HeLa cells were transfected with a control vector or Myc-tagged Peli1 and hemagglutinin-tagged ubiquitin (HA-Ub) expression vectors and cultured in presence of nocodazole (Figure 3F). Immunoblotting analyses using an immunopurified BubR1 complex demonstrated that the expression of Myc-Peli1 led to the significant polyubiquitination of BubR1. However, this polyubiquitination was almost undetectable in the Myc-epitope control. Next, the purified His-tagged Peli1 protein promoted *in vitro* polyubiquitination of the GST-fused BubR1 protein only when E1, E2, and HA-Ub enzymes were present in the reaction (Figure 3G). However, the polyubiquitination of GST-BubR1 was not induced in absence of the His-Peli1 protein. To verify the K48 and/or K63-mediated ubiquitination of BubR1 by Peli1, we transfected the expression plasmids encoding HA-K48Ub or HA-K63Ub in combination with Myc-Peli1 and Flag-BubR1 expression plasmids into HeLa cells (Figure 3H). Overexpression of Peli1 strongly elevated the level of K48-mediated polyubiquitination of BubR1 but slightly augmented those of K63-mediated polyubiquitination of BubR1. Importantly, the overexpression of full-length Peli1 (amino acids 1–418) clearly induced the appearance of a high molecular mass species of BubR1 polypeptides, a polyubiquitinated form of BubR1, whereas the overexpression of Peli1 RING domain deletion mutant (amino acids 1–280) did not do so (Figure 3I). Taken together, these results suggest that Peli1 is capable of directly regulating K48-mediated polyubiquitination of BubR1, leading to degradation of BubR1.

### Peli1 expression allows abnormal mitotic cells to adapt and become aneuploid cells

**Figure 4:**
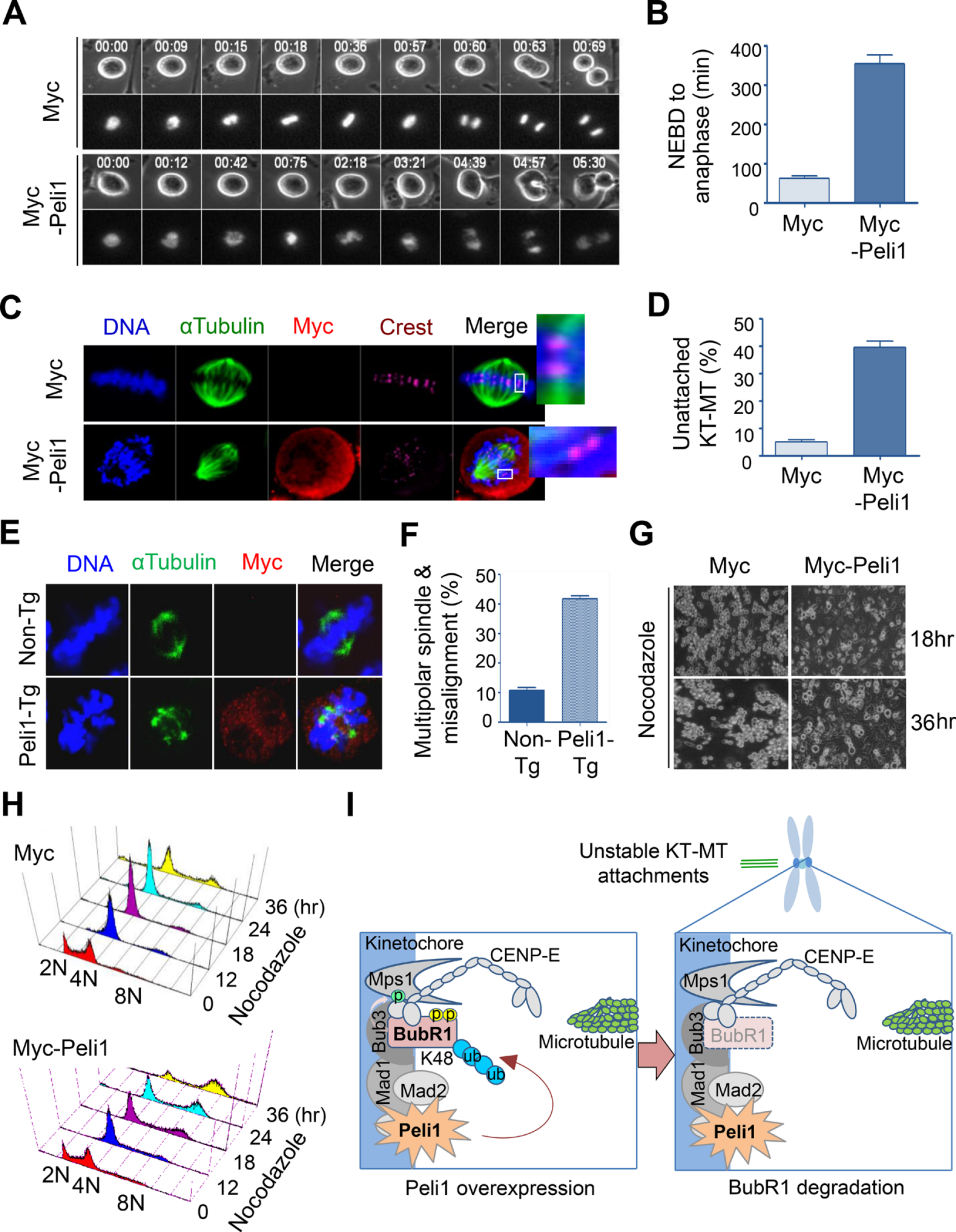
Peli1 overexpression leads to failure of kinetochore-microtubule interaction and lack of accurate chromosome alignments (**A**) HeLa cells were transfected with Myc or Myc-Peli1 and RFP-H2B (for visualization of mitotic chromosomes). The transfected cells were cultured and mitotic progression was visualized by time-lapse microscopy. Peli1 overexpressing cells showed severe delays in the metaphase-to-anaphase transition compared to control cells. (**B**) Average time from mitotic onset (nuclear envelope breakdown) to anaphase of Peli1 overexpressing cells or control cells (control cells, *n* = 24; Myc-Peli1-transfected cells, *n* = 20). (**C**) HeLa cells were transfected with a Myc (control) or a Myc-Peli1 expression plasmid. At 36 hr post-transfection, cells were subjected to a cold-stable microtubule assay and visualised by confocal microscopy. Insets show kinetochores with or without attached microtubules. Metaphase cells were visualised by staining with anti-α-tubulin (green), anti-Myc antibody (red), CREST serum (purple), or Hoechst stain (blue). (**D**) Values represent the mean percentages of cells showing unattached kinetochore-microtubule (KT-MT) interaction (> 200 cells per experiment). (**E**) Representative immunofluorescence images of non-Tg (control) splenocytes and Peli1-Tg splenocytes stained with anti-α-tubulin antibody (green), anti-Myc antibody (red), or Hoechst stain (blue). (**F**) Values indicate the mean percentages of multipolar spindles and chromosome misalignments in non-Tg and Peli1-Tg splenocytes (>100 splenocytes were counted). (**G**) Images of apoptotic HeLa cells expressing Myc (control) or Myc-Peli1 after nocodazole treatment for 18 or 36 hr. (**H**) HeLa cells were transfected with a Myc or Myc-Peli1 expression plasmid and cultured in absence or presence of nocodazole. Cells were analysed through with flow cytometry for cell cycle information. (**I**) A proposed model on how Peli1 inhibits BubR1-mediated mitotic spindle checkpoint.

We compared the timing of mitotic cell cycle progression in Peli1-overexpressing and control cells. Importantly, cells overexpressing Peli1 showed severe delays in the metaphase-to-anaphase transition with the average time from mitotic onset (nuclear envelope breakdown) to complete chromosome separation was about 340 min in Peli1-overexpressing cells, compared to about 65 min in control cells (Figure 4A, 4B). This indicated that Peli1 expression interferes with mitotic progression.

The defect in BubR1 function has recently been found to inhibit the stability of kinetochore-microtubule (KT-MT) interactions [18, 26, 27]. To analyze the effect of overexpression of Peli1 on KT-MT interactions, HeLa cells were transfected with a control backbone vector or a Myc-Peli1 expression plasmid, and the presence of cold-stable KT-MT interactions was examined as previously described [28, 29]. Overexpression of Peli1 had considerable influence on KT-MT interactions and led to failure of accurate chromosome alignment at the metaphase plate (Figure 4C, 4D). This indicated that the overexpression of Peli1 reduced KT-MT stability. In addition, splenocytes derived from non-Tg and Peli1-Tg mice were immunostained with anti-αTubulin and anti-Myc antibodies (Figure 4E, 4F). Of note, the Peli1-overexpressing splenocytes (43.5%) showed much larger increases in multipolar spindle formation and subsequent chromosome misalignment than control splenocytes (11.6%). Interestingly, aneuploidy formation in Peli1-overexpressing splenocytes was also induced without additional mitotic defects, such as seen with microtubule inhibitors or with mutational inactivation of mitotic checkpoint genes (data not shown).

Because overexpression of Peli1 led to a sharp decrease in BubR1 stability and a considerable destabilisation of KT-MT interactions, we examined the effect of Peli1 expression on the frequency of chromosomal aneuploidy in cells subjected to mitotic spindle damage. HeLa cells were transfected with control vector or Myc-Peli1 expression vector and then treated with nocodazole (Figure 4G, 4H). Control-transfected cells exhibited mitotic arrest at 36 hr after treatment, and then cells gradually underwent apoptotic cell death (Figure 4G, left panels and Supplementary Figure 2E). The Peli1-overexpressing cells subjected to spindle damage, however, overrode mitotic arrest and produced a significant aneuploid cell population with relatively few apoptotic cells (Figure 4G, right panels and Supplementary Figure 2E). The extended incubation of Peli1-overexpressing cells in the presence of nocodazole led to the marked accumulation of aneuploid cells. Flow cytometry analysis further confirmed this result (Figure 4H). HeLa cells overexpressing Peli1 significantly accumulated their aneuploid population compared to control-vector transfected cells, indicating that Peli1 expression allows abnormal mitotic cells to adapt and become aneuploid.

Overall, these results indicate that Peli1 expression produced mitotic dysfunction and chromosomal aneuploidy by ubiquitin-mediated degradation of BubR1 and thus contributed to neoplastic aneuploidy transformation.

## DISCUSSION

Peli1 as part of a family of signal-responsive ubiquitin E3 ligases is required for TLR and TCR signalling as well as for production of several proinflammatory cytokines and maintenance of self-tolerance. However, homozygous *PELI 1* knockout mice have no overt abnormalities in growth or survival [1]. Importantly, although Peli1 mRNAs are ubiquitously expressed in most organs, the proteins are present at very low levels in many human cell types. In addition, Peli1 is overexpressed in the majority of patients with aggressive B cell lymphoma, and Peli1 expression appears to be closely associated with poor prognosis among B cell lymphoma patients [6]. This suggests that a gain-of-function for the *PELI 1* gene may be a causative pathogenic step. Presently, a gain-of-functional study of Peli1 was conducted *in vitro* and in *vivo*. The results indicated that Peli1 expression promotes the ubiquitinational degradation of BubR1 and that this leads to severe mitotic defects (Figure 4I), resulting in marked chromosomal aneuploidy and tumorigenesis. Overt phenotypes occurring in Peli1-overexpressing mice were very similar to those reported to occur in BubR1-insufficient mice [19, 20].

Mitotic checkpoint proteins play an important role in preventing abnormal mitotic cells with chromosomal aneuploidy from achieving neoplastic aneuploidy, but it remains unclear how BubR1 signalling is regulated during the neoplastic transformation. In addition, no study has found an inactivating mutation of BubR1 in human cancers. However, recent studies have suggested that the BubR1 protein is regulated by post-translational modifications such as acetylation, phosphorylation, sumoylation, and ubiquitination [24, 26, 30–32]. Although these modifications influence the function and stability of BubR1, their physiological relevance remains to be completely established. In this regard, this paper is the first to describe the effects of inhibitory signalling on BubR1-mediated chromosome integrity and establishes the *in vivo* relationship between failure of BubR1 upstream signalling and tumour development.

In the present study, Peli1 expression mediated the ubiquitination and degradation of BubR1 *in vivo* and *in vitro*. BubR1 has also been reported to contain a destruction box motif, which is required for ubiquitin-dependent proteolysis [24]. BubR1 is physically associated with the multisubunit E3 ubiquitin ligase APC/C [12, 14] and is periodically degraded during the transition from mitosis to interphase. These results suggest that the undesirable ubiquitination or degradation of BubR1 may be an important pathogenic mechanism underlying aneuploidy and tumorigenesis. However, this raises the important question of how the ubiquitinational degradation of BubR1 is governed during tumorigenesis. Presently, the levels of BubR1 were markedly lower in cells expressing Peli1 *in vitro* and *in vivo*, suggesting that the ubiquitin E3 ligase activity of Peli1 may specifically influence the susceptibility of BubR1 to ubiquitin-dependent proteolysis (Figure 4I). In support of this hypothesis, we also identified that the levels of Peli1 protein were highly elevated, particularly in a number of cells from cancer patients, such as aggressive B cell lymphomas [6]. These observations imply that the forced degradation of BubR1 by over-expression of Peli1 may allow formation of chromosome aneuploidy and thus tumorigenesis. However, the transient expression of Peli1 in HeLa cells reduced the levels of hyperphosphorylated BubR1 but not those of the hypophosphorylated form, which suggests that the ubiquitinational degradation of BubR1 may be phosphorylation-dependent. In support of the above hypothesis, previous studies have reported that certain types of ubiquitin ligases, such as the SKP1-CUL1-F-box protein ubiquitin ligase, target phosphorylated substrates for proteasomal degradation [33].

A partial loss of the mitotic checkpoint function is not inherently lethal but can cause severe mitotic damage, as shown by BubR1 and Mad2 knockdown cells. However, the forced expression of Peli1 is a more potent promoter of acquisition of aneuploidy and mitotic failure in nocodazole-treated cells (> 70% of the cells affected) than in BubR1 or Mad2 knockdown cells (~60% affected) [21, 25, 34]. In general, aneuploidy caused by mitotic defects occurs in the context of additional defects, such as spindle damage. Presently, however, the expression of Peli1 generated severe aneuploidy even in the absence of additional mitotic defects or spindle damage. Therefore, it is conceivable that the overexpression of Peli1 is a potent driver for acquisition of chromosomal aneuploidy and subsequent tumorigenesis.

Because it is possible that the expression of Peli1 can lead to loss or gain of genes associated with the mitotic cell cycle, cell survival, or apoptosis, the aberrant expression of Peli1 during development may be lethal due to the high probability of chromosome instability and severe mitotic defects being created. Indeed, transgenic mice expressing high levels of the Peli1 transgene had significantly shorter life spans, which is consistent with the proposition that chromosomal aneuploidy is closely related to lethality in mice [35–37]. These findings may explain why Peli1 levels are extremely low and barely detectable in most cells.

Apoptotic cell death plays an important role in preventing chromosomal aneuploidy from evolving into neoplastic aneuploidy. Interestingly, previous studies have found that the exogenous expression of BubR1 induces apoptosis and inhibits expansion of tumours established with BubR1-depleted aneuploid cells in mice [24] and that BubR1 triggers the intrinsic apoptotic pathway upstream of caspase-9, thereby activating caspase-3 [24, 38, 39]. These results indicated that the formation of aneuploidy following sustained mitotic checkpoint activation appears to be proceeded by the ubiquitin-dependent proteolysis of BubR1. Therefore, the adaptation into acquisition of preneoplastic aneuploidy may be functionally connected to the BubR1-mediated apoptosis of unstable aneuploid cells. In this work, Peli1 expression was able to override the apoptotic cell death following prolonged mitotic arrest induced by microtubule inhibitor treatment. These results suggest that Peli1 antagonises the apoptotic activity of BubR1 through ubiquitinational degradation and augmented aneuploidy after prolonged mitotic arrest.

It is important to identify the signalling pathways responsible for the regulation of Peli1 expression as they can provide new insights into the molecular lesions underlying tumorigenesis. In particular, Peli1 expression may lead to constitutive activation of antiapoptotic NFκB signalling (possibly in conjunction with TCR-CD28, TLR-IL1R, and/or BCR-CD40R signalling) [1, 4, 5, 40], which raises the possibility that Peli1 expression may be an initiating oncogenic stimulus for evolution of neoplastic aneuploidy and tumour cell survival. It is also possible that Peli1-induced aneuploidy generates a second gain-of-function pathway involving activation of an oncogene or an anti-apoptotic gene (e.g., BCL2 and BCL6) and the mutation of multiple Ig and non-Ig genes, among others. In this regard, future research should examine the relationships between Peli1 and pathogenic conditions, such as lymphomas, and determine how Peli1 is differentially expressed in different tissues and cell types. Nevertheless, our results suggest that Peli1 expression acts as an inhibitory signal in the homeostatic regulation of the mitotic cell cycle and its checkpoints by inducing the ubiquitination-mediated BubR1 degradation, and thus contributes to the initiation and progression of neoplastic chromosome aneuploidy.

## MATERIALS AND METHODS

### Generation and genotyping of peli1 transgenic mice

For generation of transgenic mice expressing the human Peli1 coding sequence, a Peli1 expression vector was constructed with a beta-actin promoter and a human cytomegalovirus (CMV) early enhancer to induce the ubiquitous expression of Peli1 transgene in most tissues. For facilitation of transgene detection, a Myc-epitope tag was included upstream of the Peli1 coding sequence. The vector was linearized, purified, and injected into the pronuclei of fertilised C57BL/6J mice. Polymerase chain reaction (PCR) and Southern blotting showed that 3 of 14 pups derived from the quadruple embryo transfer were positive for the transgene. Transgenic Peli1 transcripts were PCR-amplified by using primers specific to the transgenic fusion product: Myc 5′-GACGAATTCCC GCGGATGGAGCAGAA-3′ (forward) and Peli1 5′-GACC TCGAGAGGTCGTGCTGCATTGATT-3′ (reverse). All animal experiments were conducted in accordance with the guidelines set by the Institutional Animal Care and Use Committee (IACUC) of Sungkyunkwan University School of Medicine (SUSM), which is accredited by the Association for Assessment and Accreditation of Laboratory Animal Care International (AAALAC International) and abides by the Institute of Laboratory Animal Resources (ILAR) guidelines.

### Histology and immunoblotting

Tissues were fixed in 10% neutral buffered formalin overnight at room temperature, dehydrated and embedded in paraffin. Tissues were 3-μm sectioned and stained with hematoxylin and eosin (H&E) and microscopic images were captured using an AxioCam digital microscope camera and AxioVision image processing software (Carl Zeiss). Tissues and transfected cells were then lysed in a buffer solution (150 mM NaCl, 20 mM HEPES, 5 mM EDTA, 0.5% Nonidet P-40, 1 mM PMSF, 10 mM NaF, 1 mM Na_3_VO_4_, 1 mM DTT, and protease inhibitors), and lysates were separated by SDS-PAGE and analysed by immunoblotting using anti-Myc, anti-BubR1, anti-Peli1, and anti-actin antibodies.

### Immunofluorescence and microtubule stability experiments

HeLa cells were grown on poly-L-lysine-coated cover slips, fixed with 5% formaldehyde for 10 min, washed with PBS containing 0.1% Triton X-100, and blocked for 1 hr in PBS containing 0.1% Triton X-100 and 3% BSA. The cells were then incubated with the indicated antibodies, stained with Hoechst, and monitored under an LSM500 META confocal microscope (Carl Zeiss). For cold-stable microtubule attachment, transfected cells were treated with Monastrol for 5 hr and then released by treating them with MG132 (a proteasome inhibitor) for 1 hr. The resulting metaphase-arrested cells were incubated in L-15 medium (Invitrogen) containing 20 mM HEPES (pH 7.3) on ice and then fixed at room temperature for 10 min in 3.7% formaldehyde in 100 mM PIPES (pH 6.8), 10 mM EGTA, 1 mM MgCl2, and 0.2% Triton X-100. After fixation, the cells were blocked in PBS containing 0.1% Triton X-100 and 3% BSA and then incubated with the indicated antibodies. DNA was visualised by DAPI staining.

### MEF and splenocyte cultures and karyotype analyses

Primary MEFs were obtained from mouse embryos at E13.5 days and cultured in DMEM supplemented with 10% FBS (Hyclone). For metaphase spreads from MEFs, 1.5 × 10^6^ cells were treated with 0.05 μg/ml colcemid (Gibco BRL) for 6 hr, and for metaphase spreads from splenocytes, spleens were freshly collected and minced between two microscope slides. The released cells were then suspended in 5 ml PBS, centrifuged at 1,000 rpm for 5 min, resuspended in 4 ml of RPMI 1640 supplemented with 10% FBS, IL-2 (10 U/ml), PHA (5 μg/ml), ConA (5 μg), and colcemid (0.05 μg/ml), and further cultured for 6 hr. After treatment, MEFs and splenocytes were harvested, suspended in 5 ml 0.075 M KCl, and incubated for 30 min. Finally, the cells were fixed in Carnoy's solution (75% methanol and 25% acetic acid), stained with Giemsa, and analysed under the LSM500 META confocal microscope (Carl Zeiss).

### Flow cytometry

For the cellular DNA analysis, the cells were harvested at various time points, fixed in 70% ethanol, and stained with 40 μg/ml of propidium iodide (PI) in presence of 50 μg/ml of RNase A. The DNA content of 10,000 cells/sample was analysed. Data acquisition was performed using the FACSCanto II flow cytometer (BD Biosciences).

### *In vitro* binding and immunoprecipitation assays

For the GST pull-down assays, fusion proteins were adsorbed onto glutathione-protein A/G-Sepharose beads (Amersham Biosciences) and incubated with whole-cell extracts from asynchronised or nocodazole-treated HeLa or Ramos cells. Bound proteins were separated by 8% sodium dodecyl sulfate-polyacrylamide gel electrophoresis (SDS-PAGE) and analysed by immunoblotting with an anti-BubR1 or anti-Myc antibody. For immunoprecipitation, transfected cells were treated with or without 200 ng/ml nocodazole for 18 hr, resuspended in an immunoprecipitation buffer solution (150 mM NaCl, 20 mM HEPES, 5 mM EDTA, 0.5% Nonidet P-40, 1 mM phenylmethanesulfonylfluoride, 10 mM NaF, 1 mM Na_3_Vo_4_, and 1 mM dithiothreitol supplemented with a mixture of protease inhibitors), and incubated at 4 °C for 30 min. The cells were then lysed by passing the cell clumps five times through a 27-gauge needle. The lysates were centrifuged at 13,000 rpm for 30 min, and the insoluble fraction was discarded. Lysates were incubated with an anti-Myc, anti-flag, anti-BubR1 antibody or normal IgG (control) and then with protein A/G agarose beads, which were later pelleted, washed three times with immunoprecipitation buffer, and analysed by immunoblotting.

### *In vitro* kinase assay

Transfected cells were lysed, and BubR1 was immunoprecipitated from lysates. Pelleted beads were washed with the immunoprecipitation buffer and then three times with the kinase buffer [20 mM HEPES (pH 7.8), 15 mM KCl, 10 mM MgCl2, 1 mM EGTA, and 0.1 mg/ml BSA] and then reacted with 20 μg of histone H1 in presence of unlabelled ATP or 20 μCi [γ^32^P]ATP at 30^°C^ for 30 min. The reaction was stopped by adding SDS sample buffer. Proteins were resolved by 8% SDS-PAGE, and the results were quantified by autoradiography. For the *in vitro* phosphorylation of GST-BubR1, 1 μg of GST-BubR1 was washed twice with kinase buffer [20 mM HEPES (pH 7.8), 15 mM KCl, 10 mM MgCl2, 1 mM EGTA, and 0.1 mg/ml BSA] and reacted with 400 ng of recombinant Cdk1/Cyclin B1 or Plk1 (both from Invitrogen) in presence of unlabelled ATP (0.2 mM) at 30°C for 1 hr. Phosphorylated GST-BubR1 proteins were incubated with 5 μg His-Peli1 for 5 hr at 4^°^C. Bead-bound protein complexes were washed twice with the immunoprecipitation buffer and analysed by immunoblotting.

### *In vivo* and *in vitro* ubiquitination assays

HeLa cells were transfected with an expression plasmid encoding Myc or Myc-tagged Peli1 or Peli1 deletion mutants, Flag-tagged BubR1 and HA-tagged ubiquitin (HA-Ub) in the indicated combinations. The cells from each plate were collected into two aliquots. One aliquot was used for conventional immunoblotting, and the remaining cells were used for immunoprecipitation of the BubR1 protein complex. Immunoprecipitates were washed three times with TNN buffer, and bound proteins were immunoblotted with the indicated antibodies. Purified GST or GST-BubR1 (200 ng) was incubated with purified His-Peli1 (1 μg) in conjunction with E1 (50 ng UBE1, Boston Biochem), E2 (400 ng UncH13/Uev1a, Boston Biochem), and HA-tagged ubiquitin (2 μg HA-Ub, Boston Biochem) in ubiquitin reaction buffer [5 mM Tris-HCl (pH 7.5), 2 mM MgCl_2_, 2 mM ATP and 100 mM NaCl]. Reaction mixtures were incubated for 2 hr at 37 °C and analysed by immunoblotting with anti-BubR1 or anti-ubiquitin and anti-Peli1 antibodies.

### Cell fractionation

Asynchronized or synchronized cells were lysed with fractionation buffer 1 (10 mM Tris, 25 mM KCl, 5 mM MgCl2, 0.5% NP-40, 1 mM dithiothreitol, 1 mg/ml aprotinin, 1 mg/ml leupeptin, 5 mM NaF and 0.5 mM Na_3_VO_4_) and incubated on ice for 10 min. The supernatant fraction was collected by centrifugation at 13,200 rpm for 10 min at 4^°^C. Then a 2^nd^ supernatant fraction was used as a soluble cytosolic fraction. The insoluble pellet was washed twice with 0.5 M NaCl and lysed with fractionation buffer 2 (10 mM Tris, 500 mM NaCl, 0.1% NP-40, 5 mM EDTA, 1 mg/ml aprotinin, 1 mg/ml leupeptin, 5 mM NaF and 5.5 mM Na_3_VO_4_) by sonication. After centrifugation at 13,200 rpm for 5 min at 4^°^C, the chromatin-bound nuclear fraction was obtained.

### Statistical analysis

In all experimental data, the error bars represent mean ± S.D. or S.E.M from at least three independent experiments. Student's test was used for statistical comparisons.

## SUPPLEMENTARY MATERIALS FIGURES


