# Supplementary material for: Pellino 1 inactivates mitotic spindle checkpoint by targeting BubR1 for ubiquitinational degradation
Source: Oncotarget. 2017 Mar 31;8(19):32055–67. doi: 10.18632/oncotarget.16762 (PMC5458268; doi:10.18632/oncotarget.16762)
Supplement: Supplementary file 1 [file oncotarget-08-32055-s001.pdf]

Pellino 1 inactivates mitotic spindle checkpoint by targeting BubR1 for ubiquitinational degradation

Supplementary Materials

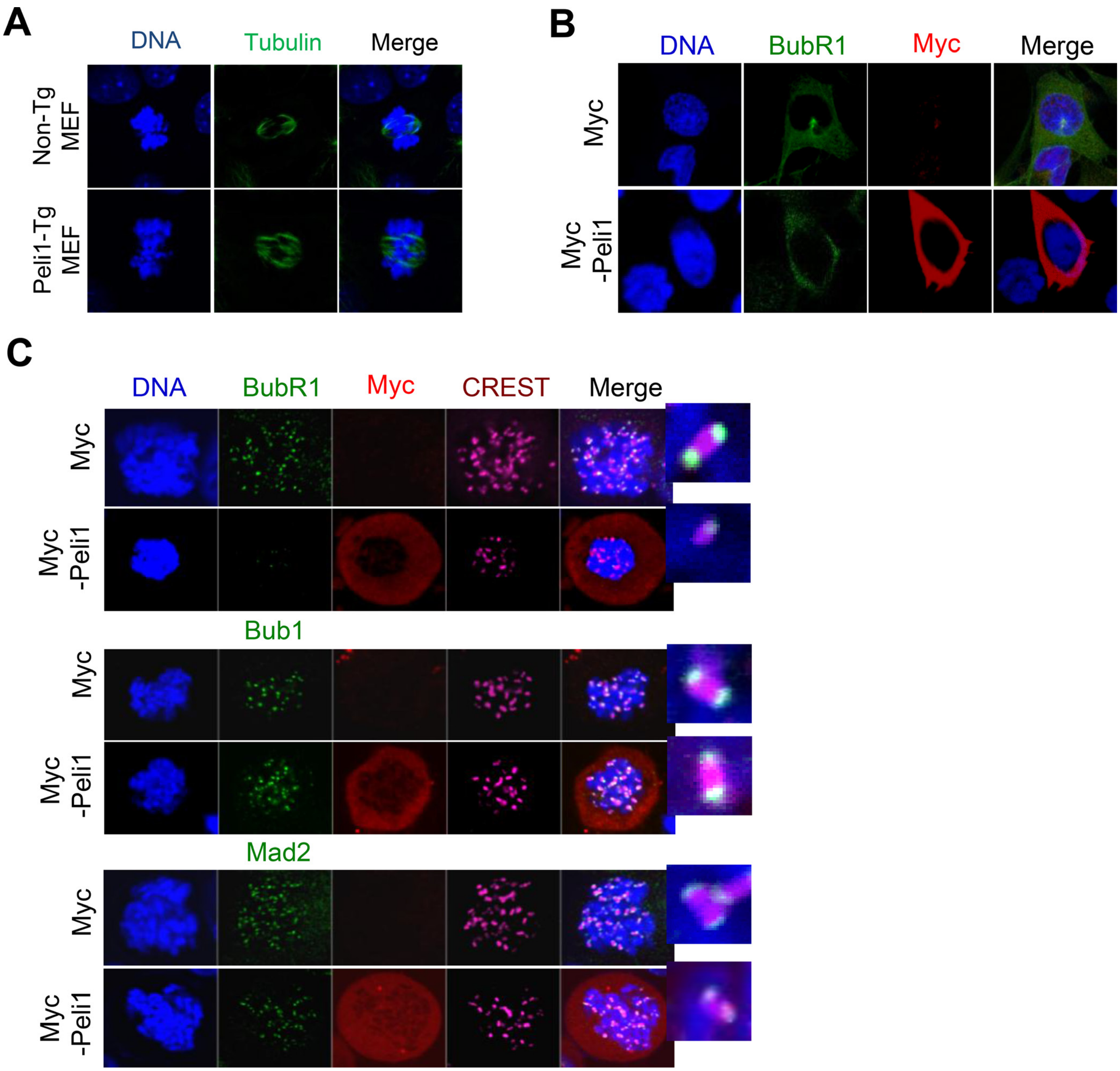

Supplementary Figure 1: Peli1 specifically inhibits the stability of the BubR1 mitotic checkpoint protein. (**A**) Imaging of MEFs of Non-Tg and Peli1-Tg for nuclear staining. These were stained with anti-Hoechst antibody (blue) and anti-Tubulin antibody (green). (**B**) HeLa cells were transiently transfected Myc or Myc-Peli 1 expressionplasmid and asynchronized cells were stained with anti-BubR1 (green) and anti-Myc (red) and Hoechest (blue). (**C**) HeLa cells were transiently transfected with a Myc or Myc-Peli 1 expression plasmid. After nocodazole treatment, synchronized cells were stained with an anti-BubR1 or Bub1 or Mad2 (green), an anti-Myc antibody (red), the CREST serum (purple), or Hoechst (blue), and examined by confocal microscopy.

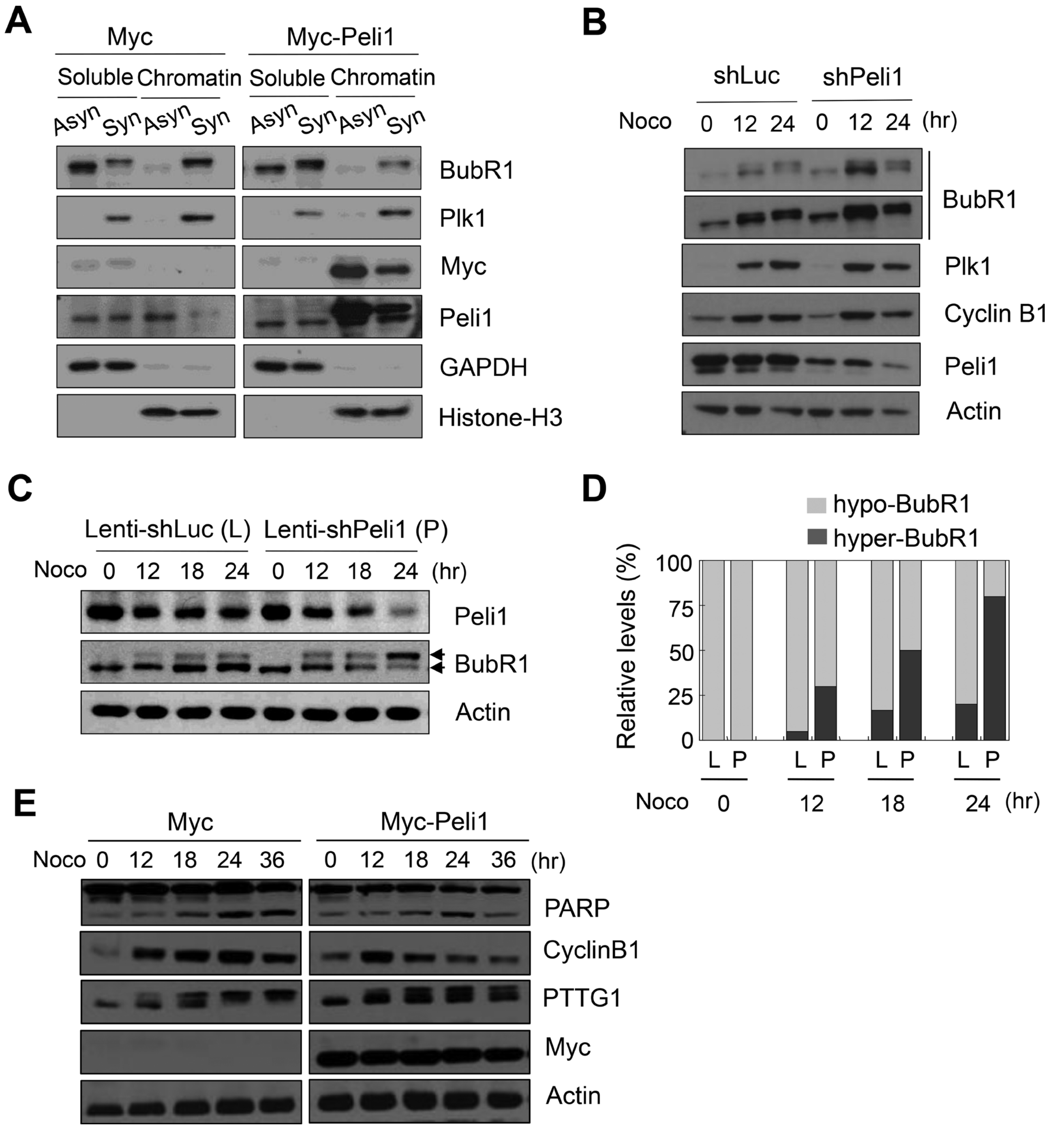

Supplementary Figure 2: Peli1 overexpression decreases hyperphosphorylation and kinetochore association of BubR1. (**A**) Chromatin fractions of asynchronized and synchronized by nocodazole HeLa cells were obtained. Proteins were eluted as soluble fractions and chromatin-bound fractions were analyzed by immunoblotting with the indicated antibodies. Histone-H3 and glyceraldehydes-3-phosphate dehydrogenase were used as controls for chromatin and soluble fractions, respectively. (**B**) HEK293 cells were transfected with shluc (control) or shPeli1 (knock down of Peli1) plasmids. At 24 hours after transfection, cells were treated with nocodazole for 12 or 24 hours and harvested for immunoblotting with anti-BubR1, Plk1, cyclinB1, Peli1, or actin antibodies. (**C**) Ramos cells were infected with lentivirus expressing shRNA of luciferase (Lenti-shLuc, L) or Peli1 (Lenti-shPeli, P). Cells were treated with nocodazole for different time-points and harvested for immunoblotting with anti-BubR1 antibody. (**D**) The graph shows the relative amounts of hyperphosphorylated (upper arrow) and hypophosphorylated (lower arrow) BubR1 proteins, based on the results presented in (C). (**E**) HeLa cells were transfected with a Myc or Myc-Peli1 expression plasmid, at 24 hr post-transfection, treated with nocodazole for different time-points. Harvested cellular lysates were immunoblotted with an anti-p*oly (ADP-ribose) polymerase* antibody.
